# Fecal microbiota transplantation in patients with slow-transit constipation: A randomized, clinical trial

**DOI:** 10.1371/journal.pone.0171308

**Published:** 2017-02-03

**Authors:** Hongliang Tian, Xiaolong Ge, Yongzhan Nie, Linfeng Yang, Chao Ding, Lynne V. McFarland, Xueying Zhang, Qiyi Chen, Jianfeng Gong, Ning Li

**Affiliations:** 1 Department of General Surgery, Jinling Hospital, Medical School of Nanjing University, Nanjing, China; 2 Institute of Digestive Diseases, Xi Jing Hospital, Fourth Military Medical University, Xi’An, China; 3 BGI-Shenzhen, Shenzhen, China; 4 Department of Medicinal Chemistry, University of Washington, Seattle, Washington, United States of America; 5 Shanghai Tenth People's Hospital, Tenth People's Hospital of Tongji University, Shanghai, China; University Hospital Llandough, UNITED KINGDOM

## Abstract

Fecal microbiota transplantation has been proposed as a therapeutic approach for chronic constipation. This randomized, controlled trial aimed to compare the effects of conventional treatment alone (control) with additional treatment with FMT (intervention) in patients with slow-transit constipation (STC). Adults with STC were randomized to receive intervention or control treatment. The control group received education, behavioral strategies, and oral laxatives. The intervention group was additionally provided 6 days of FMT. The primary endpoint was the clinical cure rate (proportion of patients achieving a mean of ≥ three complete spontaneous bowel movements [CSBMs] per week]. Secondary outcomes and safety parameters were assessed throughout the study. Sixty patients were randomized to either conventional treatment alone (n = 30) or FMT (n = 30) through a nasointestinal tube. There were significant differences between the intervention group and control group in the clinical improvement rate (intention-to-treat [ITT]: 53.3% vs. 20.0%, *P* = 0.009), clinical cure rate (ITT: 36.7% vs. 13.3%, *P* = 0.04), mean number of CSBMs per week (ITT: 3.2 ± 1.4 vs. 2.1 ± 1.2, *P* = 0.001), and the Wexner constipation score (ITT: 8.6 ± 1.5 vs. 12.7 ± 2.5, *P* < 0.00001). Compared with the control group, the intervention group showed better results in the stool consistency score (ITT: 3.9 vs. 2.4, *P* < 0.00001) and colonic transit time (ITT: 58.5 vs. 73.6 h, *P* < 0.00001). The intervention group had more treatment-related adverse events than did the control group (50 vs. 4 cases). FMT was significantly more effective (30% higher cure rate) for treatment of STC than conventional treatment. No serious adverse events were observed.

## Introduction

Fecal microbiota transplantation (FMT), also known as fecal bacteriotherapy or fecal infusion, consists of injection of a liquid filtrate of feces from a healthy donor into the gastrointestinal tract of a recipient individual [1]. FMT has been proposed as a therapeutic approach for functional diseases of the gastrointestinal tract by reestablishment of a wide diversity of intestinal flora.

Numerous case reports, retrospective case series, and randomized, controlled trials have shown the benefit of FMT in patients with functional bowel disorders, including inflammatory bowel disease and irritable bowel syndrome [2–4]. Slow-transit constipation (STC) is defined as an increased colonic transit time (CTT) as measured by radionucleotide techniques or radiopaque markers. Patients with STC respond poorly to an increase in dietary fiber and have variable responses to laxatives [5]. Decreased colonic motility is an important pathophysiological mechanism of STC. Recently, several studies have suggested that gut microbiota may be involved in the etiology of constipation. Imbalance in composition of stool microbiota has been described in patients with constipation [6–9].

Our previous study demonstrated that reestablishment of intestinal microbiota was directly linked to improvement of intestinal motility in patients with STC [10]. Borody et al. theorized that significant relief of constipation by FMT was due to restoration of intestinal homeostasis [11]. However, no systematic, large studies have evaluated the efficacy of this treatment. Therefore, we performed a randomized, single-blind, placebo-controlled, clinical trial to assess the efficacy and safety of FMT for STC.

## Materials and methods

### Study population

Patients who were diagnosed as STC and were not responsive to traditional treatments with diet modification [12], enemas, or biofeedback in the previous 6 months were enrolled from November 2015 to February 2016. Eligibility criteria included the following: age > 18 years; body mass index of 18 to 25 kg/m^2^; and STC defined as a CTT of > 48 h [13–15]. This clinical trial (registered at ClinicalTrials.gov: NCT02526849) was a randomized, single-blind, clinical study that was undertaken at Jinling Hospital. Jinling Hospital is a tertiary center that treats a large number of patients with chronic constipation in China.

### Ethics statement

Our study protocol was reviewed and approved by the Institutional Ethics Review Board at Nanjing University Jinling Hospital (2015NZKY-020). All experiments were performed in accordance with the Declaration of Helsinki. All of the patients were asked to provide written, informed consent before participating and for the 12-week follow-up period from December 2015 to May 2016.

### Trial design

A randomization list for 60 patients with an allocation ratio of 1:1 was generated without stratification according to a computer-generated random number. Randomization was performed by the opaque sealed envelope system, in which the study nurse randomly opened a preformed envelope containing the allocated treatment regimen. The investigators were blinded to the treatment assignment. Patients were not blinded to the treatment.

### Control group

These constipated patients were advised to participate in a conventional treatment during the 12-weeks study period. Conventional treatment consisting of education, behavioral strategies, and oral laxatives, and they also avoided any other probiotics during the 12-weeks study period [16], was taken by both groups.

If patients did not have a bowel movement for 3 or more consecutive days, they were permitted to take up to 20 g of macrogol 4000 powder (Forlax^®^, Ipsen, Paris, France). If ineffective, an enema could be used.

### Intervention group

On days 1–6, patients received 100 ml fresh FMT by a nasointestinal tube, once per day. The nasointestinal tube was placed in the patient’s proximal jejunum through endoscopy. The donor fecal microbiota was then infused within 5 min through the nasointestinal tube. During the 12-week study period, these patients combined FMT with conventional therapy.

### Outcome measures and endpoint

In this study, we compared the efficacy and safety of FMT compared with controls in patients with STC. The primary outcome measure was the clinical cure rate (proportion of patients with an average of 3 or more CSBMs per week during the 12-week follow-up). The primary endpoint is 12 weeks after treatment.

The secondary efficacy outcomes included the following. (1) The clinical improvement rate was defined as the proportion of patients with an average increase of one or more CSBMs per week, but they did not meet the diagnostic criteria for a clinical cure or patients with a cure. (2) The number of CSBMs per week was recorded. (3) The CTT was measured at baseline and at week 12 using the Metcalf method. Briefly, a capsule containing radio-opaque markers (small barium strip, 22 mm, Nanjing Hospital of Traditional Chinese Medicine) was taken orally with breakfast every day for 6 consecutive days. The CTT was calculated based on the number of markers that were detained in the colon by abdominal X-ray after 24 h administration of the final dose of markers. [13,15] (4) Subjective assessment of stool consistency was documented at every telephone follow-up call. The pattern of stool consistency was categorized based on the Bristol Stool Form Scale [17]. Stools that were rated as 1 or 2 on the Bristol Stool Form Scale were defined as hard, those rated 6 or 7 were defined as loose, and those rated 3, 4, or 5 were defined as normal. (5) The Wexner constipation scale is a validated and internationally adopted questionnaire, which is used to quantify the severity of constipation [18]. This scale consists of questions examining the various clinical expressions of constipation, with scores ranging from 0 (best) to 30 (worst). The Wexner constipation scale was evaluated before and 12 weeks after the intervention. (6) Safety endpoints were measured by the frequency of adverse events (defined as development of any gastrointestinal symptoms [e.g., abdominal pain, diarrhea, nausea, vomiting, bloating, and flatulence]) or any other side effects during FMT and the follow-up period.

No interim analysis was planned or performed, and no early stopping rules were implemented. All of the patients maintained a daily bowel symptom diary. The length of follow-up was 12 weeks.

### Donor screening

Eligible donors could be an intimate, long-time partner, friend, or unrelated volunteer. Donor exclusion criteria included the following: (1) a history of antibiotic treatment during the 3 months preceding donation; (2) a history of intrinsic gastrointestinal illnesses; and (3) metabolic syndrome, obesity, or any ongoing diseases. For the trial, we used one universal donor for our trial, who was a 24-year-old healthy university student. For the purposes of informed consent, the donor had to be older than 18 years of age. Current guidelines recommend using a donor questionnaire that is similar to current protocols for screening blood donors [19]. Blood collection, including a complete blood count, chemistry, and iron profile, was performed before the FMT donation. Donor blood was negative for common viruses (hepatitis A, B, and C, HIV-1 and HIV-2, cytomegalovirus, Epstein-Barr, Herpes simplex, and *Varicella zoster*) and *Treponema pallidum*. Donor feces were negative for common enteric pathogens (Yersinia spp., Salmonella spp., Shigella spp., *Campylobacter jejuni*, *C*. *difficile* toxin, helminths, ova, parasites, and *Helicobacter pylori*), as determined with standard screening methods. The volunteer who met the selection criteria donated stool samples on the day of a patient’s FMT in the laboratory of Jinling Hospital.

### Preparation of FMT

Fresh stool (100 g) was immediately mixed in a blender with 500 mL of 0.9% sterile saline for several seconds until it developed a smooth consistency. The stool suspension was then filtered through a gauze pad to remove large particles. This suspension was then filtered several times through a decreasing number of gauze screens (2.0 to 0.7–0.2 mm) to remove small particles that could clog the nasointestinal tube. The resulting concentrated fecal bacteria suspension was administered to the patient immediately or amended with glycerol to a final concentration of 10%. This suspension was stored frozen at −20°C for 1 to 4 weeks until later use. The stool suspension was poured into a sterile bottle and administered within 2 h. This study used standardized, processed stool from the same universal donor and the same amount of stool for FMT.

### Sample size

For this study, a 15% response rate for the conventional treatment group was estimated, and we expected a 20% difference in the proportion of success between control and intervention. We calculated the total sample size based on a type 1 error of less than 0.05 and a power of 0.8. The estimated dropout rate was 10%, and a minimal sample size of 60 with 30 patients in each group was required.

### Statistical analysis considerations

Absolute and relative frequencies were calculated for qualitative variables in all patient groups. Continuous data are presented as mean±SD (M±SD), whereas categorical data are presented as n (%). Pearson’s chi-square test or Fisher’s exact test for categorical variables was used, as appropriate. A *P* value < 0.05 was considered to be statistically significant. All data were analyzed using SPSS Version 20.0 (SPSS, Chicago, IL, USA).

## Results

**Fig 1:**
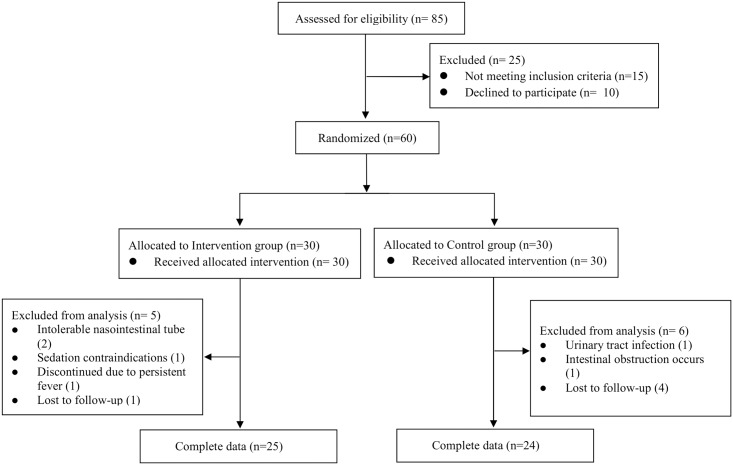
CONSORT flow diagram.

Recruitment of patients is shown in Fig 1. From November 2015 to February 2016, 85 patients were screened, 30 had conventional treatment alone (control) and 30 recipients were given conventional treatment along with FMT using stools obtained from one universal donor. All consecutive patients meeting the study inclusion criteria were offered enrollment. In intention-to-treat (ITT) analyses, 60 patients were included (30 in the FMT group and 30 in the control group). Of the 60 patients, six patients in the control group were excluded from analysis. Among these patients, four were lost to follow-up, one had a urinary tract infection, and one had intestinal obstruction. Five patients in the FMT group were excluded from analysis. Among these patients, one was lost to follow-up, two did not tolerate the nasointestinal tube, one had contraindications for sedation, and one discontinued treatment because of persistent fever. A total of 11 (18.3%) patients did not complete the allocated treatment. The rest of the 49 patients completed clinical follow-up, and were included in the per protocol (PP) analysis (25 patients in the FMT group and 24 in the control group). Three-month follow-up was completed in May 2016. There were no significant differences in the characteristics of the two study groups at enrollment (Table 1).

**Table 1:** Comparison of patients’ characteristics and history of constipation for 60 enrolled patients with slow-transit constipation.

	Control group(N = 30)	Intervention group (N = 30)	*P Value*
Age at study entry (mean S.E.)	55.4±12.1	53.1±10.2	0.425
Male: Female (n)	9:21	11:19	0.580
BMI	21.4±2.1	22.1±1.5	0.171
Disease Duration (y)	8.9±4.0	9.5±2.4	0.469
No. BM Per Week During Constipation	1.8±0.7	1.5±0.8	0.122
Wexner constipation score	13.7±2.7	14.1±2.3	0.505
Stool consistency score (BSFS score)	2.3±1.1	2.1±1.0	0.638
Straining score	3.2±0.8	3.5±0.5	0.120
Adequate of therapeutic effect of previous treatment for constipation, n (%)	8(26.7%)	6(20%)	0.540

Data are expressed as mean ± standard deviation for continuous variables, and categorical variables are expressed as absolute frequency (%) (for group).

### Primary outcome

In the FMT group, the cure rate (ITT: 36.7% vs.13.3%, *P* = 0.04) were significantly better compared with the control group (Table 2). There were also significant differences when PP analysis was performed only using the completed subjects.

**Table 2:** Treatment efficacy during the 12-week observation period: Control vs. FMT groups (intention-to-treat [ITT] and per-protocol [PP] analysis).

	ITT analysis			PP analysis		
	Control group(N = 30)	Intervention group (N = 30)	*P Value*	Control group(N = 24)	Intervention group (N = 25)	*P Value*
Clinical cure rate (%)	13.3% (4/30)	36.7% (11/30)	0.04	8.3% (2/24)	36.0% (9/25)	0.03
Clinical improvement rate (%)	20.0% (6/30)	53.3% (16/30)	0.009	20.8% (4/24)	56.0% (14/25)	0.006
Number of CSBMs per week	2.1 ± 1.2	3.2 ± 1.4	0.001	2.2 ± 0.5	3.5 ± 1.7	0.0003
Stool consistency score	2.4±1.1	3.9±1.3	<0.00001	2.5±0.8	4.2±1.9	<0.0001
Colonic transit time (h)	73.6±8.7	58.5±9.8	<0.00001	71.5±10.6	56.4±7.5	0.01
Wexner constipation score	12.7±2.5	8.6±1.5	<0.00001	11.3±0.8	8.4±1.2	0.0002

### Secondary outcomes

Five secondary clinical outcomes were significantly improved in the FMT group compared with the control group (Table 2). These outcomes included the clinical improvement rate (ITT: 53.3% vs. 20.0%, *P* = 0.009), the number of CSBMs per week (ITT: 3.2 ± 1.4 vs. 2.1 ± 1.2, *P* = 0.001), the stool consistency score (ITT: 3.9 ± 1.3 vs. 2.4 ± 1.1, *P* < 0.00001), the CTT (ITT: 58.5 ± 9.8 vs. 73.6 ± 8.7 h, *P* < 0.00001), and the Wexner constipation score (ITT: 8.6±1.5 vs. 12.7±2.5 h, *P* < 0.00001). There were also significant differences when PP analysis was performed only using the completed subjects, with a significant change in these five secondary clinical outcomes (Table 2).

### Safety

Adverse events are shown in Table 3. The most frequently reported adverse events were mild or moderate in severity and were transient, including venting, nausea, abdominal pain, and diarrhea. Endoscopy-related respiratory difficulty (22 vs. 0 cases) and nausea (12 vs. 0 cases) were more prevalent in the FMT group than in the control group. These symptoms disappeared after the tube was removed when treatment was finished. The incidence of these adverse events was similar between the two groups in the follow-up period. Except for the events described above, there were no more adverse events during the FMT procedure and the follow-up period.

**Table 3:** Adverse events that were observed during the 12-week observation period.

	Control group (N = 30)	Intervention group (N = 30)
No of adverse events (times)	4	50
Sedation contraindications	0	1
Endoscopy related respiratory difficulty	0	22
Nausea	0	12
Abdominal pain	3	5
Diarrhea	0	4
Flatulence	1	4
Transient fever	0	2

## Discussion

In this randomized, controlled trial, FMT was more effective than conventional treatment (education, behavioral modification, oral laxatives, probiotics, and rescue with macrogol), in terms of efficacy for the treatment of constipation. This efficacy was based on outcomes, such as the clinical improvement rate, clinical cure rate, frequency of patients’ mean number of bowel movements per week, and the Wexner constipation score. We found better stool consistency scores and colonic transit times for FMT compared with conventional treatment. In contrast, patients who had conventional treatment had fewer adverse events than those who had FMT.

The primary endpoint of an average of three or more CSBMs per week was chosen because it indicates normal bowel function and constipation. In 36.7% of patients, constipation disappeared after FMT, but constipation disappeared only in 13.3% of the control group. More than half (53.3%) of patients in the FMT group had an average increase of one or more CSBMs per week. This improvement was accompanied by a faster transit, and a more significant improvement in CTT. These findings support the hypothesis that the efficacy of FMT on constipation is at least partly dependent on its effects on colonic motility. Recently, Vandeputte et al. [20] observed a strong association between stool consistency and gut microbiota richness and composition. Parthasarathy et al. [21] found that the profile of the fecal microbiota, especially genera from *Firmicutes* (*Faecalibacterium*, *Lactococcus*, and *Roseburia*), was correlated with colonic transit.

Most of the adverse events in the FMT group were related to the method of nasointestinal tube delivery. In the FMT group, 22 patients felt uncomfortable and had respiratory difficulty when the nasointestinal tube was inserted. Patients in the FMT group also reported more common mild to moderate adverse events, such as fever, venting, and flatulence, than did those in the control group. This may have been due to the availability of suspended microbial metabolites and associated increased immune response in the fecal suspension. Most of the adverse events disappeared after 6 days of FMT treatment. Currently, there is no clear consensus on the best route of fecal microbiota administration, which includes via colonoscopy, enema, and a nasojejunal or nasogastric tube [22]. According to a meta-analysis by Kassam et al., efficacy did not differ via the nasojejunal or colonscopy route [23]. In the current study, we chose the nasojejunal route because it is easy to retain the donor stool suspension and eliminate the necessity of repeated endoscopy. Youngster et al. significantly simplified the clinical practice of FMT [24]. They removed the need for invasive gastrointestinal procedures using acid-resistant hypromellose capsules, which contained stool suspensions. We hypothesized that fecal microbiota could be further freeze-dried, such as probiotics orally, and self-administrated at home [25]. FMT is reported to be effective by all of these routes, and the preferred method may vary with the clinical situation. We believe that the profound interest in FMT reflects the perception that it is a safe treatment for STC. Therefore, patients appear willing to try FMT, but how willing physicians in various specialties are to offer, perform, or refer patients for this treatment is unknown.

This study has several limitations. First, the follow-up was limited to 12 weeks after the last FMT, which is insufficient to evaluate the long-term safety of two treatments. However, this time frame was longer than the majority of clostridium difficile infection therapeutic trials, which typically ranged from 2 to 8 weeks [26]. Second, this was a single-center study, and the sample size of this trial was relatively small. Third, more comprehensive evaluation of the intestinal microbiome needs to be performed, such as using 16S ribosomal RNA and whole-genome shotgun sequencing. We have collected the samples to perform this study and intestinal microbiome analysis is underway.

In the future, the complex preparation of fecal suspension for FMT should be standardized when designing the protocol. There is also a need to improve delivery methods to increase the accessibility and acceptability of this type of treatment.

## Conclusions

In adults with STC, 6 days of FMT increase CSBMs per week, soften stool, speed up transit, and improve symptoms of constipation, with a cure rate of 30% higher than conventional treatment. FMT is a promising cure in 1/3 of adult patients with STC. Optimizing treatment could result in further improvement.

## Supporting information

S1 Text Ethics approval document. (DOCX)

S2 Text Study protocol. (DOC)

S3 Text Consort 2010 checklist. (DOC)

S4 Text Original dataset. (XLS)
