# Supplementary material for: Fecal microbiota transplantation in patients with slow-transit constipation: A randomized, clinical trial
Source: PLoS One. 2017 Feb 3;12(2):e0171308. doi: 10.1371/journal.pone.0171308 (PMC5291446; doi:10.1371/journal.pone.0171308)

Ethics approval (English version)

| Title | Fecal Microbiota Transplantation in Patients with Slow Transit Constipation: study protocol for a randomized controlled trial | | |
| --- | --- | --- | --- |
| Applying Department | Department of General Surgery, Jinling Hospital | | |
| Research/Department | Li Ning/ Jinling Hospital | | |
| Category | Clinical trial | Apply Year | 2015 |
| Inspector | Liu Yuxiu | | |
| Items | 1. Check table 2. Revised version 3. Clinical protocol(Version 3.0,2015.10.20) 4. Consent table(Version 4.0,2015.10.20) | | |
| Review of comments / requests  1. any change in the course of the study program, reporting needed;  2. serious adverse events, the timely submission of serious adverse events report;  3. according to the review frequency, regular submission of research reports;  4. if there is violation of the test program, the need to submit a timely report;  5. the researchers suspended or early termination of clinical research, timely submission of reports;  6.when the test is completed, researcher should submit the report | | | |
| Check frequency | 12 months | | |
| Chief signature | Liu Yuxiu | | |
| Date | 10.21.2015 | | |
| Facility | Institutional Ethics Review Board of Jinling hospital | | |

Address of Institutional Ethics Review Board of Jinling hospital: Zhongshan East Road 305, Nanjing, Jiangsu, China. Tele:025-80863234

Ethics approval (Chinese version)


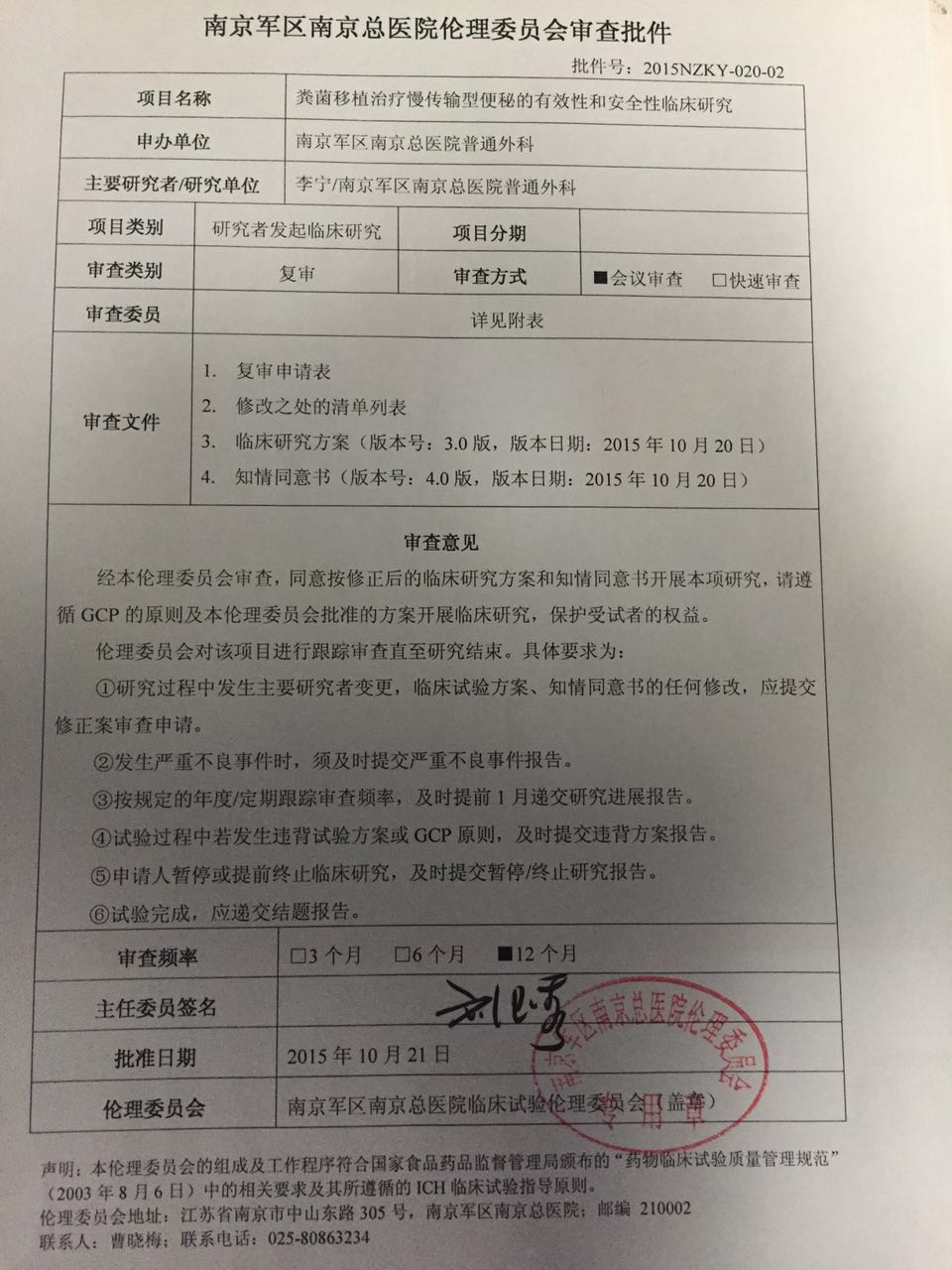

Supplement: S1 Text — (DOCX) [file pone.0171308.s001.docx]
