# Supplementary material for: Fecal microbiota transplantation in patients with slow-transit constipation: A randomized, clinical trial
Source: PLoS One. 2017 Feb 3;12(2):e0171308. doi: 10.1371/journal.pone.0171308 (PMC5291446; doi:10.1371/journal.pone.0171308)
Supplement: S2 Text — (DOC) [file pone.0171308.s002.doc]

**Study Protocol**

| Name | | Fecal Microbiota Transplantation in Patients with Slow Transit Constipation: study protocol for a randomized controlled trial | | | | |
| --- | --- | --- | --- | --- | --- | --- |
| Applying Department | | Department of General Surgery, Jinling Hospital | | | | |
| Version | | 3.0 | | Date | | 2015.10.21 |
| **Content** | | | | | | |
| **Contents**  FMT has been proposed as a therapeutic approach for functional diseases of the gastrointestinal tract by reestablishment of the wide diversity of intestinal flora. Slow-transit constipation（STC）is defined as decreased colonic transit as measured by a radionucleotide techniques or radiopaque markers. Patients with STC respond poorly to an increase in dietary fiber and have variable responses to laxatives. Decreased colonic motility is an important pathophysiological mechanism of STC. Recently, several studies suggested that gut microbiota involved in the etiology of constipation. Imbalance in stool microbiota composition was described in patients with constipation. However, there are no systematic large studies to evaluate the efficacy of this treatment. A randomized, single-blind, placebo-controlled clinical trial was therefore performed to assess the efficacy and safety of FMT for STC.  **Inclusion Criteria**  Chronic constipation according to Rome III criteria, defined as two or fewer spontaneous, complete bowel movements (CSBMs) per week for a minimum of 6 months; Age ≥ 18 years; BMI: 18.5-25 kg/m2; Slow colonic transit confirmed by colonic transit test (colonic transit time (CTT) > 48 hours); Normal anorectal manometry, with no evidence of dyssynergia and confirmed ability to expel rectal balloon; No radiographic evidence of functional (i.e. pelvic floor dyssynergia) or anatomical (i.e. significant rectocele and intussusception) impediment to the expulsion of the radio-opaque contrast; Disease duration > 1 year;  Traditional treatment with dietary modification, laxatives (including osmotic and stimulant laxatives), and biofeedback tried over the past 6 months without success;  **Exclusion Criteria**  Bowel constipation due to innate factor (i.e. megacolon) or secondary interventions (i.e. drugs, endocrine, metabolic, neurologic or psychologic disorders); History or evidence of gastrointestinal diseases (i.e. obstruction, cancer, inflammatory bowel diseases) ; Previous abdominal surgery, except cholecystectomy, appendicectomy, tubal ligation and cesarean section;  Previous proctological or perianal surgery; A constipation condition meeting the Rome III criteria for IBS or functional abdominal pain syndrome; Pregnant or breast-feeding women; Infection with enteric pathogen; Usage of probiotics, prebiotics and/or synbiotics within the last month; Usage of antibiotics and/or PPIs within the last 3 months; Smoking or alcohol addiction within the last 3 months; Uncontrolled hepatic, renal, cardiovascular, respiratory or psychiatric disease; Disease or therapy with drugs (i.e. antidepressants, opioid narcotic analgesics, anticholinergics, calcium antagonists, nitrates, antimuscarinics) that, in the opinion of the investigator, could affect intestinal transit and microbiota.  **Trial design**  Randomized Controlled Trial  **Control group**  These constipated patients were advised to participate in a conventional treatment during the 12-weeks study period. Conventional treatment was taken by both of two groups. If patients did not have a bowel movement for 3 or more consecutive days, they were permitted to take up to 20 g of Macrogol 4000 powder (Forlax®, Ipsen, Paris, France). If ineffective, an enema could be used.  **Intervention group**  On day 1-6, patients received 100ml fresh FMT by nasointestinal tube, once per day. The nasointestinal tube was placed in the patient’s proximal jejunum through endoscopy. Then, donor fecal microbiota was infused within 5 minutes through nasointestinal tube, add conventional treatment (consisted of education, behavioral strategies, and oral laxatives, and to avoid any other probiotics during the 12-weeks study period was taken by both of two groups)  **Endpoints**  **Primary outcome**  (1) Clinical cure rate (proportion of patients with an average of 3 or more SCBMs/week during the 12 weeks follow-up);  **Secondary outcomes**  (2) Clinical improvement rate (proportion of patients with an average increase of 1 or more spontaneous complete bowel movements (CSBMs) per week) or patients with cure;  (3) Number of bowel movement per week (number of CSBMs within 1 week);  (4) Safety endpoints measured by frequency of adverse events [defined as development of any gastrointestinal symptoms (abdominal pain, diarrhea, nausea, vomiting, bloating, and flatulence, etc.)] or any other side effects during the FMT and follow-up period.  (5) The CTT was calculated based on the number of markers that were detained in the colon by abdominal X-ray after administration of the final dose of markers.  (6) Subjective assessments of stool consistency was documented at every telephone follow up call. The pattern of stool consistency was categorized based on the Bristol Stool Form Scale;  (7) The Wexner constipation scale is a validated and internationally adopted questionnaire used to quantify the severity of constipation  (6) Safety endpoints were measured by the frequency of adverse events (defined as development of any gastrointestinal symptoms [e.g., abdominal pain, diarrhea, nausea, vomiting, bloating, and flatulence]) or any other side effects during FMT and the follow-up period.  No interim analysis was planned or performed, and no early stopping rules were implemented. All of the patients maintained a daily bowel symptom diary. The length of follow-up was 12 weeks.  **Sample size**  For this study, a 15% response rate for the conventional treatment group was estimated, and we expected a 20% difference in the proportion of success between control and intervention. We calculated the total sample size based on a type 1 error of less than 0.05 and a power of 0.8. The estimated dropout rate was 10%, and a minimal sample size of 60 with 30 patients in each group was required.  **Acknowledgments** Guarantor of the article: Ning Li **Funding**  This study was supported by National Nature Science Foundation of China (no. 81670493); National Gastroenterology Research Project (2015BAI13B07);  **Disclosure**  The authors have no conflicts of interest to disclose. | | | | | | |
| Signature | Li Ning | | Date | | 10.21.2015 | |

Chinese Version

| **项目** | | 粪菌移植治疗慢传输型便秘的有效性和安全性临床研究计划书 | | | | |
| --- | --- | --- | --- | --- | --- | --- |
| **申办单位** | | 南京军区南京总医院普通外科/Jinling Hospital | | | | |
| **方案版本号** | | 3.0 | | 方案版本日期 | | 2015.10.21 |
| **内容简介** | | | | | | |
| **背景简介**  近 年 来 ，粪 菌 移 植（fecal microbiota transplantation，FMT）作为一种历史悠久的重建肠道菌群疗法重新被临床所关注， 即将健康人粪便中的功能菌群， 通过一定方式移植到病人肠道内， 调节肠道菌群失衡， 重建具有正常功能的肠道微生态系统， 辅助治疗肠道内、外疾病。本研究通过开展FMT 技术治疗慢传输型便秘（slow transit constipation，STC） 病人随机对照研究， 探讨其有效性和安全性。  纳入标准  入选标准： ① 符合罗马Ⅲ标准关于慢性便秘的诊断标准； ② 年龄≥18 岁； ③ BMI: 18.5-25 kg/m2； ④ 结肠传输试验显示结肠传输时间CTT＞48h； ⑤ 排粪造影显示没有明显的功能性（如盆地失迟缓）或器质性（如直肠前突、 内 套叠）改变； ⑥ 肛管直肠测压显示排便时没有直肠病理性收缩或肛门括约肌协同运动障碍； ⑦ 病程＞1年； ⑧ 过去6个月通过饮食调节、泻剂（ 包括渗透性和刺激性泻剂） 、 灌肠、生物反 馈等常规治疗疗效不佳。 **排除标准：** ① 先天性巨结肠； ② 继发性便秘： 继发于药物、 内分泌、代谢、神经或精神等因素； ③ 胃肠疾病史， 如梗阻、肿瘤、 IBD； ④ 腹部手术史， 阑尾切除、胆囊切除、输卵管结扎、剖宫产除外； ⑤ 直肠或肛周手术史； ⑥ 符合罗马Ⅲ标准关于 IBS 或功能性腹痛的诊断标准； ⑦ 怀孕或哺乳的女性； ⑧ 肠道病原体感染的征象； ⑨ 过去 3 个月内服用抗生素或 PPIs； ⑩ 过去 1 个月内服用过益生菌、 益生元或合生元； ⑪ 过去 1 个月内吸烟或酗酒； ⑫ 肝肾、心肺疾病或精神异常； ⑬ 合并影响肠动力的疾病或服用影响肠动力的药物， 如抗抑郁药、阿片类麻醉性 镇痛药、抗胆碱能药、钙拮抗剂、硝酸盐、 抗毒蕈碱药。  **研究设计**  随机对照试验  **干预组**  符合纳入标准患者每天100ml粪菌液，共计6天，通过鼻肠管给予，联合常规的便秘治疗（饮食教育，行为干预及必要时口服泻剂12周）  标准  **对照组**  **该组便秘患者仅仅接受常规便秘治疗**（饮食教育，行为干预及必要时口服泻剂12周）  **结局指标**  **首要结局指标**   1. 临床治愈率；   次要结局指标   1. 临床改善率； 2. 每周完全排便次数； 3. 安全性指标（相关不良反应）； 4. CTT结肠传输实验； 5. 随访期间便秘的严重度评分和胃肠生活质量评分；   粪便硬度评分（ BSFS 评分， 1-7 分，分数越小，粪便越干硬）； 粪便硬度正常（ BSFS=3 或 4） 的情况所占比例； 排便的费力程度评分（ 1-5 分，分数越大， 排便越费力）； 排便不费力（评分=1 或 2） 的情况所占比例； 排便不尽感程度评分（ 1-5 分，分数越大， 排便不尽感越强）； 排便不尽感不强（评分=1 或 2） 的情况所占比例；  **样本量计算**  根据之前研究报道对照组治疗，我们预期20%的FMT组治愈率差值，同时考虑到每组10%的失访率，计算可得每组样本量各需30例；  **课题负责人**  李宁  **基金：**  国家自然科学基金（81670493）及科技部临床医学研究（消化疾病）协同网络建设示范应用研究（2015BAI13B07）  **声明**  研究参与者均无相关利益冲突。 | | | | | | |
| 申请人签字 | 李宁 | | 日期 | | 10.21.2015 | |
